# Plasmid-Mediated Co-Occurrence of *mcr-1.1* in Extended-Spectrum *β*-Lactamase (ESBL)-Producing *Escherichia coli* Isolated From the Indigenous Seminomadic Community in Malaysia

**DOI:** 10.1155/2024/9223696

**Published:** 2024-10-09

**Authors:** Polly Soo Xi Yap, Li-Fang Yeo, Cindy Shuan Ju Teh, Amreeta Dhanoa, Maude Elvira Phipps

**Affiliations:** ^1^Jeffrey Cheah School of Medicine and Health Sciences, Monash University Malaysia, Bandar Sunway 47500, Subang Jaya, Selangor, Malaysia; ^2^Cancer Research Malaysia, Sime Darby Medical Centre Subang Jaya, 2nd Floor, Outpatient Centre, Subang Jaya 47500, Selangor, Malaysia; ^3^Department of Medical Microbiology, Faculty of Medicine, Universiti Malaya 50603, Kuala Lumpur, Malaysia

**Keywords:** antimicrobial resistance, colistin resistance, extended-spectrum *β*-lactamase *Escherichia coli*, *mcr-1* gene, remote indigenous community, rural health

## Abstract

The growing prevalence of commensal antibiotic resistant *Escherichia coli* poses a significant concern for the global spread of antibiotic resistance. Stool samples (*n* = 35) from a seminomadic indigenous community in Malaysia, the Jehai, were screened for multidrug-resistant bacteria, specifically the extended-spectrum *β*-lactamase (ESBL) producers. Subsequently, whole-genome sequencing was used to provide genomic insights into eight ESBL-producing *E. coli* that colonised eight individuals. The ESBL *E. coli* isolates carry resistance genes from various antibiotic classes such as the *β*-lactams (*bla*_TEM_, *bla*_CTX-M−15_ and *bla*_CTX-M−55_), quinolones (*gyrA*, *qnrS* and *qnrS1*) and aminoglycosides (*aph*(*3′*)*-Ia*, *aph*(*6*)*-Id* and *aac*(*3*)*-IId*). Three concerning convergence of ESBL, colistin and metal resistance determinants, with three plasmids from H-type lineage harbouring *bla*_CTX-M_ and *mcr-1.1* genes were identified. Using the Oxford Nanopore Technology (ONT) Native Barcoding Kit (SQK-NBD114.24) in conjunction with the R10.4.1 flow cell, which achieved an average read accuracy (*Q* > 10) of 99.84%, we further characterised the *mcr-1.1*-bearing plasmids, ranging in size from 25 to 28 kb, from three strains of *E. coli*. This report represents the first whole genome analysis of multidrug-resistant bacteria, specifically those resistant to colistin, found within the indigenous population in Malaysia. It strongly indicates that the pertinent issue of colistin resistance in the country is far more significant than previously estimated.

## 1. Introduction

The indigenous people of Peninsular Malaysia, also known as the Orang Asli (OA), have their livelihood intrinsically connected to the natural environments as well as the companion animals. Due to the unique challenges of remoteness, the extent of antimicrobial resistance (AMR) affecting the OA communities has largely remained neglected. During our previous study assessing the cardiometabolic and gut health of the seminomadic Jehai community [1], a total of eight non-duplicate commensal extended-spectrum *β*-lactamase (ESBL)-producing *Escherichia coli* (ESBL-EC) were isolated and selected from the stool samples of eight unrelated individuals for whole genome analysis. The Jehai are traditionally hunter-gatherers who live deep in the rainforest of Peninsular Malaysia [2]. Nowadays, the younger Jehai prefer to seek employment in nearby townships. Hunting in the rainforest where the last of the Malayan Tigers roam along with poisonous snakes do come with certain risks. Hunting is practiced occasionally and more among the older generation [3]. The Jehai diet mainly consists of a myriad of forest greens, rice, river fish and occasionally meat (either bought or hunted).

AMR studies involving OA communities are severely limited. The AMR trends in *E. coli* isolated from OA were previously reported by Mohamed-Yousif et al. [4] and Mariappan et al. [5]. However, both studies did not investigate further on the genomic determinants of antibiotic resistant *E. coli* and correlate with their phenotypic characteristics. Factors such as close contact with animals, limited access to clean water and sanitation and traditional healing practices may contribute to increased exposure to AMR bacteria and the transmission of resistance genes. It is important to study the underlying eco-evolutionary dynamics of commensal *E. coli* among indigenous populations who live in rural areas with limited access to healthcare and presumably less exposure to the common sources of AMR such as healthcare facilities, antibiotics and farmed poultry. Moreover, given the adaptable nature of *E. coli* in acquiring resistance genes and its propensity to evolve into a nosocomial pathogen, conducting comprehensive investigations across multiple settings in South-East Asia becomes paramount.

Using a combination of short and long-read sequencing, this study undertook the genomic characterisation encompassing AMR and stress response genes, virulence factors, mobile genetic elements and phylogenetic analysis of ESBL-EC isolated from this research, along with Malaysian strains sourced from public database.

## 2. Methodology

### 2.1. Community Engagement

We engaged with the Jehai community in Royal Belum Rainforest, located in the state of Perak. Upon discussion and agreement from the village elders and headman, we returned on a preagreed date with a team of medical doctors and fieldwork scientists. We recruited participants (*n* = 35) over the age of 18 years old who could provide informed consent and had no visible ailments or disabilities. Stool samples were aliquoted into 15% glycerol solution, transported back to Monash University Laboratory on dry ice and stored at −80°C.

### 2.2. Bacteria Isolates, Antimicrobial Susceptibility Testing and Whole-Genome Sequencing (WGS)

Stool samples (*n* = 35) were revived on Tryptic Soy Broth at 37°C for up to 48 h in a shaking incubator. A total of 200 μl stool solution was pipetted onto selective, chromogenic Brilliance ESBL (Oxoid, UK) agar plates and incubated at 37°C for 24 h. Negative plates were further incubated for an additional 24 h. Coloured colonies that presumptively suggested ESBL based on the manufacturer's instructions were further purified on MacConkey agar. Presumptive ESBL-ECs (blue/pink isolates) were recovered from 12 specimens (34% of the collected specimens). ESBL confirmatory test was conducted using double disk synergy test (DDST), using both cefotaxime (CTX) (30 μg) and ceftazidime (30 μg) alone and in combination with clavulanic acid. *Klebsiella pneumoniae* ATCC 700603 (positive control) and *E. coli* ATCC 25922 (negative control) were used for quality control for the DDST. Colonies from eight specimens (23%) were confirmed as ESBL producers. Eight non-duplicated ESBL-ECs from different individuals were then selected for further testing. Disk diffusion test was also performed using amoxicillin–clavulanate acid (20 μg/10 μg; AMC), piperacillin–tazobactam (30 μg/5 μg; TZP), aztreonam (30 μg; ATM); ceftazidime (30 μg; CAZ), cefotaxime (30 μg; CTX), cefoxitin (30 μg; FOX), cefepime (30 μg; FEP) and meropenem (10 μg; MEM; Oxoid, Thermo Scientific). All tests were performed in accordance to the Clinical and Laboratory Standards Institute (CLSI) M100 protocol, and the breakpoints were determined based on the recommended guidelines [6]. Genomic DNA of the isolates was extracted using QIAGEN DNeasy Blood and Tissue Kit (Qiagen, Hilden, Germany) according to the manufacturer's instructions. DNA quality control was measured using NanoDrop spectrophotometer (NanoDrop One; Thermo Fisher Scientific) and Qubit 3.0 Fluorometer with dsDNA HS Assay Kit (Invitrogen, Life Technologies). The library was constructed using Nextera XT DNA Library Preparation Kit (Illumina, San Diego, CA) with 150 bp paired-end reads and subsequently subjected to WGS using the Illumina NovaSeq (Illumina, San Diego, CA).

### 2.3. Quality Control, Genome Assembly, Annotation and Pan-Genome Analysis

The paired-end reads were quality filtered to remove adapters and low-quality read with quality scores <30 using Fastp (v0.20.0). Subsequently, the reads were assembled *de novo* using SPAdes (v3.15.5) with default settings. The genomes were annotated by Prokka (v1.14.5). Assembled genomes were analysed for Achtman 7 gene Multi Locus Sequence Typing (MLST; v2.0.9) [7, 8], core genome MLST (cgMLSTFinder 1.2), serotypes (SerotypeFinder 2.0) [9], mobile genetic elements (MGEfinder 1.0.3) and plasmids (PlasmidFinder 2.1) [10, 11] using the Center for Genomic Epidemiology (CGE) web tools. Resistance, virulence and stress response genes identification was performed using AMRFinderPlus (v3.11.14) [12]. The output was harmoniased and consolidated using hAMRonization (v1.1.1; https://github.com/pha4ge/hAMRonization). The phylogroup of the isolates was determined by EzClermont (v0.6.3; https://github.com/nickp60/EzClermont).

### 2.4. Phylogenetic Analysis

*Escherichia coli* isolates from Malaysia that were available on EnteroBase (https://enterobase.warwick.ac.uk/) with full assembled genomes (*n* = 24) were downloaded and included for phylogenetic analysis. The genome sequences were aligned using the reference sequence alignment-based phylogeny (REALPHY; v1.13) via bowtie2 aligning tool [13]. Maximum likelihood phylogeny of core and pangenome of 32 Malaysian *E. coli* were inferred from the alignment using FastTree (v2.1.11) with 1000 bootstraps. The resulting phylogenetic tree was visualised in combination with the metadata and the corresponding AMR genes using Microreact [14].

### 2.5. *mcr-1.1* Harbouring Plasmids Extraction and Nanopore Sequencing

Based on the whole genome characterisation, three *mcr*-plasmid-harbouring strains (JHEC01, JHEC06 and JHEC11) were identified. Each of the single colony of the isolates was inoculated into 5 mL of Luria–Bertani (LB) broth and incubated overnight at 37°C with shaking for 16 h. As previously described [15, 16], plasmid DNA was isolated using QIAGEN Plasmid Midi Kit (Qiagen, Hilden, Germany) according to the manufacturer's instructions. DNA quality and molarity were assessed using Qubit 3.0 Fluorometer with dsDNA HS Assay Kit (Invitrogen, Life Technologies) and 4200 TapeStation with Genomic DNA ScreenTape (5067–5365, Agilent Technologies). The sequencing library was prepared using Native Barcoding Kit 24 V14 (SQKNBD114.24, ONT) and sequenced using R10.4.1 flow cell (FLO-MIN114, ONT) on a MinION Mk1c device with MinKNOW v23.11.7. The high-accuracy base-calling mode was selected, the minimum Q score was set at 8, while other parameters were kept at their defaults.

### 2.6. Plasmid Assembly and Characterisation

Dorado (https://github.com/nanoporetech/dorado) was used for base calling. Assembly of MinION reads was done using an automated *de novo* assembly pipeline (https://github.com/rchapman2000/ont-de-novo-assembly) using Flye (v2.9.3; https://github.com/fenderglass/Flye) and polished with Medaka “r1041_e82_400bps_hac_g615” model. Besides, BLAST was utilised to determine the identity of the contigs and identify the *mcr*-containing plasmids in comparison to the reference genome.

The acquired AMR genes were annotated using ResFinder (v4.5.0; http://genepi.food.dtu.dk/resfinder) by applying a 98% identity threshold and a minimum overlapping length of 60%. The identification of prophage and insertion sequence (IS) elements were identified using ISFinder (https://www-is.biotoul.fr/) [17]. We displayed the best hits and annotated the transposons or IS elements to the family level in the overlapped regions. The circular image and circular comparisons between plasmids were generated using BRIG (v.0.95) [18].

## 3. Results

### 3.1. Phenotypic and Genotypic Features of the ESBL *E. coli* Isolated from the Jehai Community

The phenotypic and genomic characteristics of the ESBL-EC isolates are summarised in Table 1. All isolates were resistant to CTX, but remained susceptible to cefoxitin (FOX) and the carbapenems.

**Figure 1:**
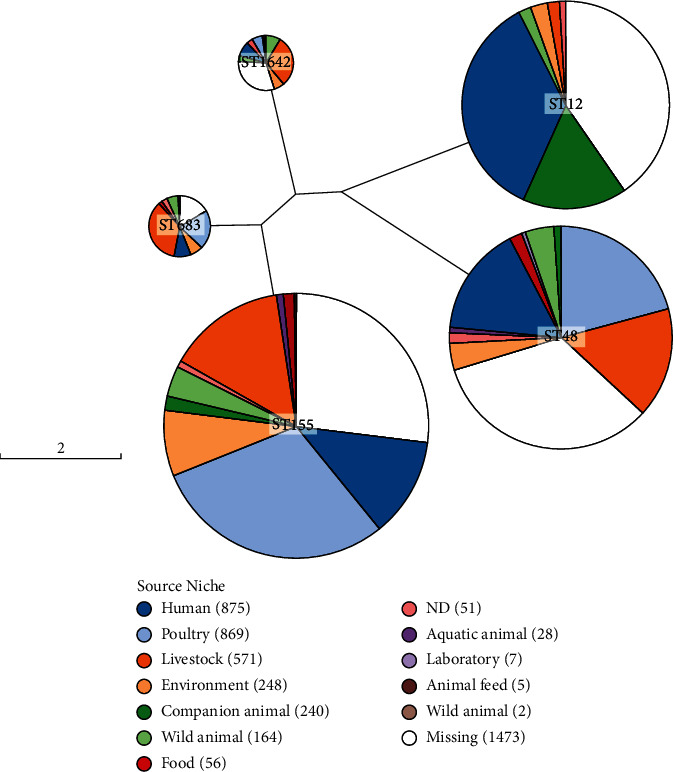
GrapeTree view showing the MLST phylogenetic relationships among the *E. coli* global strains from the EnteroBase. It consists of the five STs identified in this study.

Genome sizes, features and predicted protein-coding sequences (CDS) by Prokka annotations are shown in Table S1. Using the Achtman MLST scheme, analysis of the genomes (*n* = 8) identified five previously known sequence types (STs) and one novel ST. We observed high sequence homology based on the MLST. Half of the strains (*n* = 4) belonged to the ST155 clonal complex, suggesting that the ESBL-EC isolates had most likely arisen from the same flock of origin belonging to the same clonal complex. A search on EnteroBase for *E. coli* ST683 returned 80 hits with known sources from all over the world (including United States, Europe, China, Vietnam and Thailand). It is worth noting that majority of the isolates from the EnteroBase (71%, *n* = 57) were isolated from poultry or livestock. Only nine isolates (11%) were of human origin while the remaining isolates were isolated from environmental water (*n* = 9), wild animals (*n* = 6) and food (raw meat; *n* = 2). Similarly, ST155, ST48 and ST1642 were also dominated by poultry and livestock source niche (Figure 1). The novel ST was identified for strain JHEC06 and submitted to pubMLST for allelic assignment. The cgMLST analysis confirmed that the strain belongs to complex type (CT) 197078.

**Figure 2:**
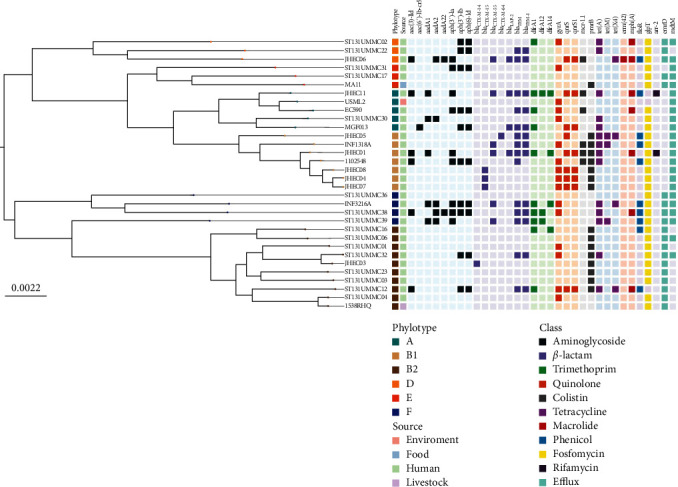
A midpoint-rooted maximum likelihood tree of Malaysian *E. coli* strains (*n* = 32) depicts the abundance of AMR genes and the interlinkage of the representative AMR gene classes across different phylogroups. A coloured box indicated the presence of AMR genes. A box with a corresponding lighter shade indicates an absence.

Following the high phenotypic *β*-lactam resistance, we identified up to four ESBL genes per isolate (JHEC01, JHEC06 and JHEC11). At the same time, these three isolates harboured the highest number of AMR genes across at least eight antibiotic classes. Following the high experimental ESBL-producing activity, we identified that the most prevalent ESBL genes were *bla*_TEM_ (*n* = 4), followed by *bla*_CTX-M−15_ (*n* = 3), *bla*_CTX-M−55_ (*n* = 3) and *bla*_LAP−2_ (*n* = 3). Notably, colistin resistant genes were prevalent, namely *pmrB* (*n* = 6) and *mcr-1.1* (*n* = 3). Additionally, quinolone resistant genes, such as *gyrA*, *qnrS* and *qnrS1*, were also present in high frequency. The absence of carbapenemase genes in our ESBL-EC strains aligns with the experimental finding of their phenotypical susceptibility to carbapenems. The highly conserved plasmid population found within these eight strains was observed. Majority (*n* = 7) of the isolates harboured at least one *F*-type plasmid, while three *mcr*-positive isolates carried *H*-type plasmids. The AMR gene profile is summarised in Figure 2.

### 3.2. The AMR Gene Profile of Malaysian *E. coli* Population is Interlinked Across Different Phylogenetic Types

To place the Jehai isolates in a local landscape, we inferred a midpoint-rooted pan-genome maximum likelihood phylogenetic tree of isolates from this study with other available Malaysian isolates and incorporated a summary of the AMR gene content (Figure 2). Majority of the Malaysian isolates were isolated from human (*n* = 21), and one each for livestock, food and environment. *E. coli* isolates from the current study were assigned to B1 (*n* = 5), A (*n* = 1), B2 (*n* = 1) and D (*n* = 1). No associations were observed between the phylogroups and AMR genes, with the exception of *emrD*, which was specific to phylogroups B2, D, E and F, while absent in phylogroups A and B1.

### 3.3. Detection of Heavy Metal Tolerance Genes Among the Jehai ESBL-EC Strains

**Figure 3:**
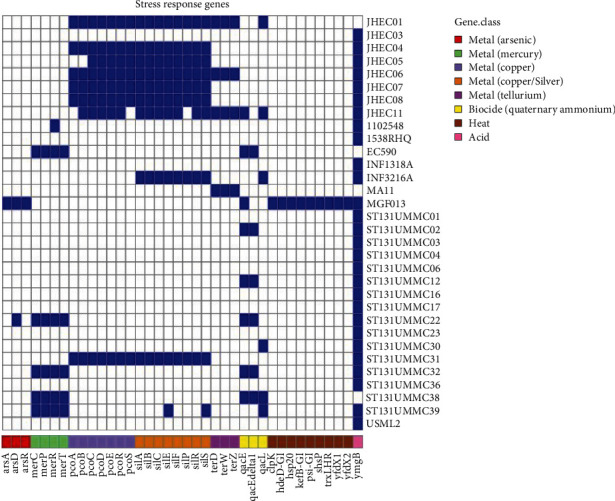
Genome wide stress response gene profile of the Malaysian *E. coli* isolates. Blue box denotes present and white box denotes absent.

Using the complete genome information, we identified the stress response genes of the Jehai ESBL-EC strains and compared the prevalence of their virulence and stress response genes with the rest of the Malaysian *E. coli* strains (Figure 3). Coexistence of silver and copper operons, *silESRCFBA* and *pcoABCDRS*, were detected for the Jehai ESBL-EC strains, except for strain JHEC03. Notably, tellurium tolerance genes (*terD*, *terW* and *terZ*) were also detected in the three genomes harbouring *mcr-1.1* (JHEC01, JHEC06 and JHEC11). In contrast, such heavy metal tolerance genes were only observed in one Malaysian clinical isolate, ST131UMMC31.

**Figure 4:**
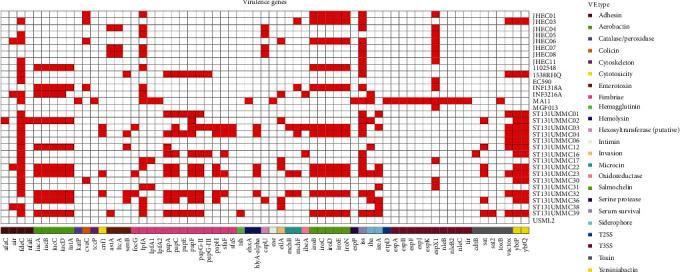
Genome wide virulence gene profile of the Malaysian *E. coli* isolates. Red box denotes present and white box denotes absent.

Contrary to the observation in genome wide stress response genes profile, the Jehai ESBL-EC isolates harboured considerably less virulence genes as compared to other Malaysian strains (Figure 4). The Jehai strains also exhibited a high homogeneity in their virulence profiles. Except for JHEC03, all isolates exhibited the presence of type III secretion system (T3SS) effector gene, *espX1*. Similarly, all isolates exhibited the serum survival gene, *iss*, except for JHEC05.

### 3.4. Genetic Characteristics of Plasmids Bearing *mcr*-1.1

The complete sequences of three *mcr-1.1*-positive IncHI2 plasmids, designated pMCR1-JHEC01, pMCR1-JHEC06 and pMCR1-JHEC11, were determined using ONT long-read sequencing (Table 2). *De novo* assembly with Flye produced a few large contigs for strain JHEC01, resulting a 281,725 base pair circular plasmid harbouring *mcr-1.1*. For strain JHEC06, the assembly revealed a 263,098 base pair plasmid containing *mcr-1.1*. strain JHEC11, which had a lower depth of coverage in nanopore reads, resulted in more fragmented assemblies. These fragments were further mapped to the most closely related plasmid, pMCR1-59496 (accession no: OP950836.1), showing 95% coverage and 100% identity.

**Figure 5:**
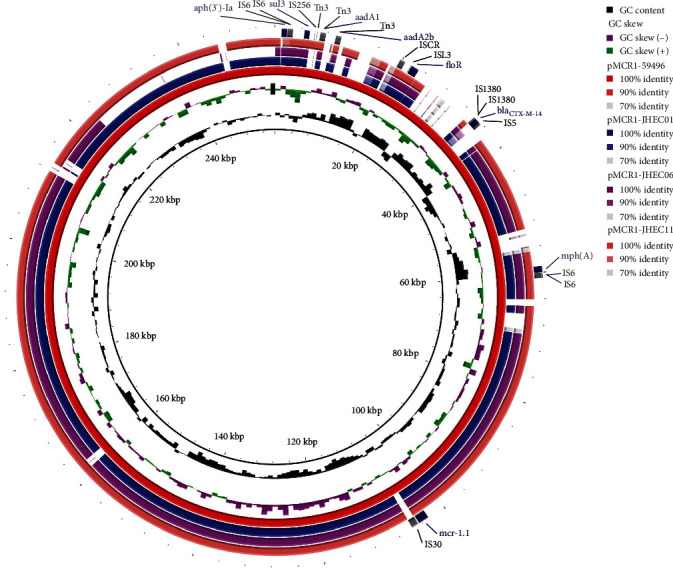
Circular comparative analysis of the *mcr1.1* bearing plasmids identified in the current study. The plasmid pMCR1-59,496 isolated from *K. pneumoniae* (OP950836.1) was used as a reference. AMR genes and insertion sequence elements were labelled at the outmost ring.

Next, we conducted multiple sequence alignment of the generated plasmid sequences. The overlapping regions of the plasmids contained segments of Tn3 family transposons, IS6 family elements and several other AMR genes, including (*aph* (*3′*)*-Ia*, *aadA1*, *aadA2b*, *sul3*, *mph* (A), *floR* and *bla*_CTX-M_), except for pMCR1-JHEC11, which lacked *bla*_CTX-M_ (Figure 5). Additionally, plasmids pMCR1-JHEC01 and pMCR1-JHEC06 harboured more AMR genes including *aac* (*3*)*-IId*, *bla*_TEM−1 B_, *bla*_LAP−2_ and *lnu* (F). Moreover, pMCR1-JHEC06 carried the tetracycline resistance gene *tet* (X4) and the quinolone resistance gene *qnrS1*.

## 4. Discussion

Our study is the first to report on whole genome of ESBL-producing *E. coli* strains originating from an indigenous community living in a remote rainforest setting. In this study, we assessed the genomic diversity of the ESBL-producing *E. coli* in terms of phylogenetic lineages, gene content, plasmid content, AMR and acquired virulence determinants. Various studies have explored the relationship between *E. coli* phylogroups with antibiotic and virulence gene patterns as well as the host species. However, there is a growing body of evidence showing that there is little indication of extensive host specificity, whereby the phylogroups are also attributed to their selective pressure and evolutionary dynamics [19]. Phylogroups A, B2 and D were often associated with human commensal, wastewater samples and pathogenic strains of *E. coli*, with significant geographical variation [20, 21]. Large-scale genomic studies indicated that phylogroup B1 was prevalent among isolates from animals and plants and carries genetic factors with environmental adaptability to soil and water reservoirs [22, 23]. The *E. coli* efflux pumps are often highly conserved [24]. Recent work by Teelucksingh et al. [25] showed that the *E. coli* efflux pumps made up 1% of the core genome. In our current study, we showed that the multidrug efflux pump gene, *emrD*, was confined within specific phylogroups (B2, D, E and F). The *emrD* is involved in the adaptive response to environmental variations [26]. The phylogroup-specific conservation of the efflux pump gene is indicative of important evolutionary trajectories of *E. coli* that predate the clinical use of antibiotics.

Increased dissemination of serum survival (*iss*) gene represents a significant and emerging clinical problem, as it is a critical factor contributing to the development of septicaemia and increased lethality rates. A previous study revealed the high conservation of the *iss* gene in virulent extraintestinal pathogenic *E. coli* (ExPEC) strains; however, the precise mechanism behind its role in enhancing serum resistance remains unclear [27]. The finding implies that *iss* likely possesses additional functions that contribute to its high conservation. In this study, the widespread occurrence of heavy metal tolerance genes among the Jehai ESBL-EC indicates the presence of selective pressure likely resulting from the co-occurrence of these compounds in nature and environmental pollution, despite the Jehai community living in a protected state park. Additionally, prevalence of the silver (*silABCDEFPRS*) tolerance genes may represent a clinically relevant concern as silver has become a popular treatment option for wound dressings [28]. Finley et al. [29] reported high prevalence of *sil* genes among the clinical isolates of 60 different species using the PCR method, of which it was predominated by *Enterobacter* (25%) and *Klebsiella* (38%). In genomic studies, the prevalence of *sil* genes has also been described among environmental and animal isolates of *Salmonella enterica* serovar and *E. coli* [30, 31]. Copper, being extensively used as a growth promoter, is among the most abundant heavy metals found in swine manure. However, when added to poultry and livestock feed, these animals absorb these additives at low rates, leading to excretion rates of up to 95% in their dung and urine and subsequently environmental pollution [32]. The high prevalence of the copper-resistance determinant (*pco*), as detected in this study, raises concerns about pollution-induced antibiotic resistance as a pressing community issue, since the possible source of copper is swine farms of which there are none, to our knowledge, in the vicinity of the community. The molecular investigation of the Pco system in *E. coli* from copper-fed swine revealed its two-operon composition: *pcoGFE* and *pcoABCDRS*, along with a single gene *pcoE* [33–35]. It is important to note that this system cannot function independently. To induce copper resistance, it relies on the activity of copper efflux from sources of copper pollution.

Based on the plasmid analysis, all the *mcr*-harbouring plasmids were IncHI2 plasmids. These findings align with prior research, demonstrating the emergence of *mcr* genes within specific Inc groups of plasmids identified in *Enterobacterales* recovered from various origins, encompassing animals, food and human subjects [15, 36–38]. While IS*Apl1* (IS30 family) was frequently observed in IncHI2 plasmids [39], such as the reference plasmid pMCR1-59496 (OP950836.1), the distribution of *mcr-1* transposons was found to be heterogeneous across and within various plasmid types [40]. In this study, we did not identify any IS elements that support the mobility of *mcr1.1*. The detection of florfenicol resistance gene, *floR*, in the plasmids co-harbouring *mcr* gene suggests extensive us of florfenicol in veterinary medicine, further accentuating the growing concern of AMR at the interface between livestock and human populations, particularly in low- and middle-income countries (LMICs) [41]. In our study, the Jehai did not raise livestock although we observed that they did keep some chickens and monkeys as pets. During our conversations with them, they appeared horrified when we asked if they ate the chickens. Hence leads to our deduction that the animals were kept as pets and not for protein. The detection of *mcr*-carrying plasmids within the OA community underscores substantial public health implications, potentially facilitating accelerated horizontal gene transfer and dissemination of colistin resistance determinants via plasmids, thus heightening the risk of resistance proliferation within the community. The compounded effect of such resistance dynamics may exacerbate the challenges in treating infections, a concern particularly pronounced for communities like OA, who reside in remote rural areas requiring extensive travel, including a 2-h boat journey, followed by an additional hour by car, to access the nearest healthcare facility.

Although our findings suggest that both animals and the environment may serve as potential reservoirs for the observed prevalence of AMR among the seminomadic Jehai community, it is important to acknowledge, in the context of One Health genomic surveillance, one of the limitations of this study: the absence of surrounding samples collected from various source niches at the point of collection. Investigating such samples would have provided a deeper understanding of the source-sink relationship of AMR, enhancing the comprehensiveness of our findings. Furthermore, the lack of representativeness of One Health samples in our genome wide phylogenetic study serves as a significant indicator, exposing the weaknesses and inadequate efforts in AMR genomic surveillance within the country.

In conclusion, our findings reveal the dissemination of ESBL-producing *E. coli* harbouring extensive classes of antibiotic resistance genes, such as colistin, *β*-lactam, aminoglycoside, fosfomycin, et cetera, from the seminomadic Jehai community. Researchers in the field have known for a long time both documented [42–44] and undocumented effects of urbanisation and deforestation encroaching on the OA lives. Our study shows evidence of intestinal carriage and dissemination of multidrug resistant bacteria in a rainforest dwelling OA community. It highlights the importance to conduct studies and implement surveillance programs specifically targeting indigenous populations to better understand the underlying factors contributing to high AMR acquisition and spread of AMR in the country. This knowledge can guide the development of tailored interventions and policies to address the unique challenges faced by these communities. This need is made more urgent by the rapidly changing urban–wildland environments of South-East Asia, with heightened risk for infectious diseases and AMR [45].

## Figures and Tables

**Table 1:** Phenotypic and genotypic characteristics of the 8 ESBL-producing *E. coli* isolates recovered from the Jehai OA faecal samples.

Strain name	Serotype	Phylogroup	ST (CC)	MLST (Achtman)	cgMLST	Antibiotic susceptibility								Plasmid type
						FEP	CTX	FOX	CAZ	MEM	AMC	TZP	ATM	
JHEC01	O100:H25	B1	155	683	78768	R	R	S	R	I	R	I	R	IncFIB, IncFII, IncHI2, IncHI2A
JHEC03	O4:H40	B2	12	12	8172	I	R	S	S	I	I	S	S	IncB/O/K/Z, IncFIB, IncFII, IncX4
JHEC04	O64:H21	B1	155	155	126227	I	R	S	S	S	S	S	I	IncB/O/K/Z, IncFIB, IncFII
JHEC05	O180:H14	B1	Singleton	1642	140670	R	R	S	R	S	R	S	R	IncFIB, IncX1, p0111
JHEC06	O84:H15	D	Singleton	14780 ^*∗*^	247824 ^*∗*^	R	R	S	R	S	I	S	R	IncFIB, IncFII, IncHI2, IncHI2A
JHEC07	O64:H21	B1	155	155	126227	I	R	S	I	S	S	S	S	IncB/O/K/Z, IncFIB, IncFII
JHEC08	O64:H21	B1	155	155	126227	I	R	S	S	S	S	S	I	IncB/O/K/Z, IncFIB, IncFII
JHEC11	O128ac:H11	A	10	48	141887	R	R	S	I	S	S	S	R	IncHI2, IncHI2A, p011

Abbreviations: AMC, amoxicillin–clavulanate acid; ATM, aztreonam; CAZ, ceftazidime; CTX, cefotaxime; FEP, cefepime; FOX, cefoxitin; I, intermediate; MEM, meropenem; R, resistant; S, susceptible; TZP, piperacillin–tazobactam.

^*∗*^Novel ST.

**Table 2:** Genomic features of plasmids bearing *mcr-1.1*.

Plasmid name	Plasmid replicon type	Size (bp)	GC content (%)	Other ARGs in *mcr* plasmids	Sequence read archive (SRA)
pMCR1-JHEC01	IncHI2	281,725	46.2	*aac* (*3*)*-IId*, *aph* (*3′*)*-Ia*, *aadA22*, *bla*_CTX-M−55_, *bla*_TEM−1 B_, *bla*_LAP−2_, *mph* (A), *lnu* (F), *floR*, *ARR-2*, *sul3*, *dfrA14*	SRR29155955

pMCR1-JHEC06	IncHI2	263,098	46.5	*aac* (*3*)*-IId*, *aph* (*3′*)*-Ia*, *aadA22*, *bla*_CTX-M−55,_*bla*_TEM−1 B,_*bla*_LAP−2,_*erm* (*42*), *mph* (A), *lnu* (F), *floR*, *qnrS1*, *sul3*, *tet* (X4)	SRR29155950

pMCR1-JHEC11	IncHI2	255,084	46.1	*aadA2*, *aadA1*, *aph* (*3′*)*-Ia*, *mph* (A), *floR*, *sul3*	SRR29155917

## Data Availability

The datasets generated during the current study are available in the NCBI SRA repository. BioProject: PRJNA990980.

## Supporting Information

Supporting Information Table S1. *Escherichia* *coli* genomes feature according to Prokka annotation.
