# Supplementary material for: Plasmid-Mediated Co-Occurrence of mcr-1.1 in Extended-Spectrum β-Lactamase (ESBL)-Producing Escherichia coli Isolated From the Indigenous Seminomadic Community in Malaysia
Source: Transbound Emerg Dis. 2024 Oct 9;2024:9223696. doi: 10.1155/2024/9223696 (PMC12017022; doi:10.1155/2024/9223696)
Supplement: Supporting Information — Table S1. Escherichia coli genomes feature according to Prokka annotation. [file 9223696.f1.docx]

**Supplementary Table. *Escherichia coli* genomes feature according to Prokka annotation.**

| Strain Name | Contigs | Size (bp) | CDS | rRNA | tRNA | tmRNA |
| --- | --- | --- | --- | --- | --- | --- |
| JHEC01 | 43 | 5,001,391 | 4097 | 9 | 65 | 1 |
| JHEC03 | 40 | 4,991,130 | 4002 | 8 | 65 | 1 |
| JHEC04 | 42 | 5,012,705 | 4113 | 9 | 67 | 1 |
| JHEC05 | 40 | 5,019,338 | 4080 | 9 | 66 | 1 |
| JHEC06 | 47 | 4,987,770 | 4132 | 9 | 67 | 1 |
| JHEC07 | 41 | 5,020,105 | 4111 | 9 | 67 | 1 |
| JHEC08 | 40 | 4,997,933 | 4104 | 9 | 67 | 1 |
| JHEC11 | 41 | 4,985,854 | 4041 | 8 | 66 | 1 |
